# Sleep characteristics and excessive daytime sleepiness in adolescents and adults: results from the birth cohorts of three Brazilian cities — RPS Consortium

**DOI:** 10.1590/1980-549720230027

**Published:** 2023-05-08

**Authors:** Susana Cararo Confortin, Iná da Silva Santos, Rosângela Fernandes Lucena Batista, Alan Luiz Eckeli, Luciana Tovo-Rodrigues, Bianca Del-Ponte, Ana Maria Baptista Menezes, Fernando César Wehrmeister, Helen Gonçalves, Viviane Cunha Cardoso, Marco Antonio Barbieri, Heloisa Bettiol, Antônio Augusto Moura da Silva

**Affiliations:** I Universidade Federal do Maranhão, Graduate Program in Collective Health – São Luís (MA), Brazil.; II Universidade Federal de Pelotas, Graduate Program in Epidemiology – Pelotas (RS), Brazil.; III Universidade de São Paulo, Faculty of Medicine of Ribeirão Preto – Ribeirão Preto (SP), Brazil.

**Keywords:** Sleep, Epidemiology, descriptive, Sleep quality, Insomnia, Excessive daytime sleepiness, Sono, Epidemiologia descritiva, Qualidade do sono, Insônia, Sonolência diurna excessiva

## Abstract

**Objective:**

To describe the prevalence of insufficient sleep duration, long sleep latency, terminal or maintenance insomnia, subjective sleep quality, and excessive daytime sleepiness among participants of birth cohorts conducted in three Brazilian cities, and to evaluate differences in prevalence rates within cohorts according to sociodemographic characteristics.

**Methods:**

Cross-sectional analyses involving adolescents and adults participating in four birth cohorts conducted in Ribeirão Preto (RP78 and RP94), Pelotas (PEL93) and São Luís (SL97/98). Sleep duration, latency, terminal or maintenance insomnia, and subjective sleep quality were obtained through the Pittsburgh Sleep Quality Index; and excessive daytime sleepiness was assessed using the Epworth Sleepiness Scale. Differences in the prevalence of the outcomes were analyzed in each cohort according to sociodemographic characteristics (skin color, marital status, socioeconomic status, study and working at the time of the interview) stratified by sex.

**Results:**

Insufficient sleep duration was the most common outcome at the four cohorts, with higher frequency among men. Long latency was more frequently reported by young adult women in RP94 and PEL93 cohorts, and insomnia by women of the four cohorts, when compared to men of the same age. Women generally suffered more from excessive daytime sleepiness and evaluated the quality of their sleep more negatively than men. In addition to sex, being a student and working were associated with the largest number of outcomes in both sexes.

**Conclusion:**

Sleep disorders are more prevalent in women, reinforcing the need for greater investment in sleep health in Brazil, without disregarding gender and socioeconomic determinants.

## RESUMO

**Objetivo:**

Descrever a prevalência de duração do sono, latência, insônia terminal, qualidade subjetiva do sono e sonolência diurna excessiva entre participantes de coortes de nascimentos realizadas em três cidades brasileiras, bem como avaliar as diferenças nas taxas de prevalência das coortes de acordo com características sociodemográficas.

**Métodos:**

Análises transversais envolvendo participantes de quatro coortes de nascimento realizadas em Ribeirão Preto (RP78 e RP94), Pelotas (PEL93) e São Luís (SL97). A duração, a latência, a insônia terminal e a qualidade subjetiva do sono foram obtidas por meio do Índice de Qualidade do Sono de Pittsburgh; e a sonolência diurna excessiva foi avaliada pela Escala de Sonolência de Epworth. As diferenças na prevalência dos desfechos foram analisadas em cada coorte segundo características sociodemográficas estratificadas por sexo.

**Resultados:**

A duração insuficiente do sono foi o desfecho mais comum nas quatro coortes, com maior frequência entre os homens. Latência longa foi mais frequentemente relatada por mulheres adultas jovens nas coortes RP94 e PEL93, e insônia por mulheres das quatro coortes, quando comparadas a homens da mesma idade. As mulheres geralmente sofriam mais com sonolência diurna excessiva e avaliavam a qualidade do sono de forma mais negativa do que os homens. Além do sexo, ser estudante e trabalhar estiveram associados ao maior número de desfechos em ambos os sexos.

**Conclusão:**

Os distúrbios do sono são mais prevalentes em mulheres, reforçando a necessidade de maior investimento na saúde do sono no Brasil, sem desconsiderar gênero e determinantes socioeconômicos.

## INTRODUCTION

Sleep can be conceptualized as a biological state that is essential for memory consolidation, as well as for the regulation of temperature, conservation and restoration of energy, and energy metabolism in the brain^1^. Sleep disturbances can affect the physical, occupational, cognitive, and social capacity of humans^1^. Furthermore, sleep disorders have been shown to negatively interfere with all systems of the human body, causing health problems such as cardiovascular disease^2^, cognitive impairment^3^ and brain deficits, and increasing the risk of neuropsychiatric disorders^4^.

Demographic and socioeconomic characteristics may be associated with different sleep domains or disorders. Studies have demonstrated a higher prevalence of long sleep latency in brown and black individuals^5,6^ and of poor sleep quality in individuals of lower socioeconomic status^7^. Furthermore, married individuals are more likely to sleep less than those who are divorced, separated, or widowed^8^. Work also affects sleep, with a higher prevalence of insomnia among people who were not working^9^. It is, therefore, necessary to understand the social and behavioral factors that are associated with a higher prevalence of different sleep domains so that guidelines, policies, and effective interventions can be formulated in order to promote health equity, as well as future actions that improve the quality of life of affected individuals.

Although many associations have been documented, few studies have assessed the prevalence of different sleep disorders and their relationship with sociodemographic characteristics. Additionally, there are no data of a sample derived from different cohorts involving different regions of the country. It is worth emphasizing the importance of describing sleep events in different cohorts and age groups, in order to verify the consistency of the results in different Brazilian regional contexts, to find out whether measures to improve sleep can be universal in the country or whether different measures would be required according to location. Therefore, the aim of this study was to describe the prevalence of insufficient sleep duration, long sleep latency, terminal or maintenance insomnia, subjective sleep quality, and excessive daytime sleepiness among participants of birth cohort studies conducted in three cities located in different regions of Brazil, and to evaluate differences in prevalence rates within cohorts according to sociodemographic characteristics.

## METHODS

### Study design

Cross-sectional data obtained from four birth cohort studies, which started in 1978 and 1994 in Ribeirão Preto (RP), in 1993 in Pelotas, and in 1997/98 in São Luís (SL), were used in the present study. The methods used for baseline sampling in each cohort and the follow-ups were previously published^10^ and are explained briefly below.

### Participants

#### Ribeirão Preto birth cohort of 1978 (RP78)

The perinatal study was conducted in eight maternity units in the municipality of RP, evaluating 6,973 children. The last follow-up occurred in 2016/2017, when 1,775 participants were evaluated at 37 to 39 years of age (sample analyzed).

#### Ribeirão Preto birth cohort of 1994 (RP94)

The perinatal study evaluated 2,911 livebirths, which corresponded to one-third of all births to mothers residing in the municipality in 1994. At 22 years of age, 1,041 young people were evaluated (sample analyzed); 622 of them belonged to the original cohort and 419 were born in RP in 1994 but had not been enrolled in the original cohort, thus adding a retrospective component to the study.

#### Pelotas birth cohort of 1993 (PEL93)

In this cohort, 5,249 livebirths that occurred in all hospitals of the municipality and whose families lived in the urban area were evaluated. So far, there have been ten follow-ups in this cohort, and the data from 3,810 subjects followed up at 22 years were used.

#### São Luís cohort of 1997/98 (SL97)

The perinatal study included one systematic sample per hospital (1/7 of births), consisting of 2,493 livebirths in ten maternity units in SL. At 18/19 years of age, 2,515 adolescents were evaluated, including 687 from the original cohort and 1,828 born in SL in 1997, who had not been selected at birth.

### Studied variables and model

#### Sleep quality

Four sleep variables, collected with questions extracted from the Pittsburgh Sleep Quality Index,^11,12^ were used: insufficient sleep duration, long sleep latency, terminal or maintenance insomnia, and subjective sleep quality. Insufficient sleep duration (<8 hours of sleep per 24 hours)^13^ was obtained by the application of the question “How many hours of sleep did you get at night?”. Long sleep latency (time taken to fall asleep ≥30 min)^14^ was collected by asking “During the past month, how long (in minutes) has it usually taken you to fall asleep each night?”. Terminal or maintenance insomnia was obtained through the question “During the past month, did you wake up in the middle of the night, at dawn or very early in the morning?”, with the following response options: “not once during the past month”, “less than once a week”, “once or twice a week”, and “three or more times a week”. Participants who responded “three or more times a week” were classified as having insomnia^11,12^. Subjective sleep quality was obtained by asking “During the past month, how would you rate your sleep quality overall?”, with the following response options: “very good”, “good”, “bad”, and “very bad”. The “bad” and “very bad” responses were classified as poor sleep quality.

### Excessive daytime sleepiness

Excessive daytime sleepiness was assessed using the Epworth Sleepiness Scale (ESS)^12,15^. The ESS contains eight questions that present situations of drowsiness in daily life, and individual answers according to the chance of falling asleep. The response options are: 0=no chance of dozing, 1=a slight chance of dozing, 2=a moderate chance of dozing, and 3=a high chance of dozing^12,15^. The ESS has a total of 24 points. In this study, participants scoring 0–10 were classified as normal, and those scoring 11 or higher as having excessive daytime sleepiness^12,15^.

### Independent variables

The following variables were evaluated: sex (male/female); self-reported skin color (white/brown/black); socioeconomic class (A-B/ C/D-E), according to the criteria of the Brazilian Association of Research Companies (ABEP in the Portuguese acronym) [A, B (B1+B2), C (C1+C2), D/E, in which A is the richest, most schooled class; and D/E, the poorest and least schooled]^16^; having a partner (no/yes); currently studying (no/yes), and currently working (no/yes).

### Data analysis

Statistical analysis was performed using the Stata 14.0 program (Stata Corporation, USA). The overall prevalence rates of insufficient sleep duration, long latency, terminal or maintenance insomnia, subjective sleep quality, and excessive daytime sleepiness were calculated for each cohort. Next, the prevalence rates for each cohort were calculated according to the independent variables. All analyses were stratified by sex, as there was a difference in most prevalences of sleep disorders by sex (Table 1). Associations were evaluated using the chi-squared test.

**Table 1:** Prevalence of insufficient sleep duration, long latency, terminal or maintenance insomnia, self-assessed poor sleep quality and excessive daytime sleepiness, according to sex in the Ribeirão Preto (1978 and 1994), Pelotas (1993) and São Luís (1997) birth cohorts. RPS Consortium, Brazil.

Cohort		RP78	RP94	PEL93	SL97
Insufficient sleep duration (%)					
	Male	77.77	71.78	70.05	54.53
	Female	70.07	61.81	56.78	56.13
	p*	0.001	0.001	0.001	0.421
Long latency (%)					
	Male	34.47	35.86	40.43	35.0
	Female	38.33	42.38	46.8	37.3
	p*	0.092	0.034	0.001	0.243
Terminal or maintenance insomnia (%)					
	Male	39.07	24.60	21.37	44.56
	Female	51.89	38.24	32.56	55.19
	p*	0.001	0.001	0.001	0.001
Subjective sleep quality (poor quality) (%)					
	Male	22.10	18.75	15.82	17.55
	Female	33.66	22.25	21.21	23.20
	p*	0.001	0.170	0.001	0.001
Excessive daytime sleepiness (%)					
	Male	25.4	23.8	38.0	23.8
	Female	28.3	32.2	62.0	32.8
	p*	0.167	0.003	0.001	0.001

* p for intra-cohort difference between sexes.

### Ethical aspects

All research projects of the birth cohorts were approved by the Ethics Committees of the respective university hospitals: Approval number 1.282.710 of the University Hospital of RP's Medical School, University of São Paulo; Approval number 1.250.366 of the Federal University of Pelotas, and Approval number 1.302.489 of the Federal University of Maranhão. The free informed consent form was signed by the participant throughout the phases of the cohorts.

## RESULTS

A total of 9,141 subjects in different stages of the life cycle were evaluated, including 1,775 from the RP78 cohort at 37/39 years of age; 1,041 from the RP94 cohort at 22 years; 3,810 from the PEL93 cohort at 22 years; and 2,515 from the SL97 cohort at 18/19 years.

In all cohorts, most participants evaluated in these follow-ups were female. Regarding skin color, most participants in the RP78, RP94 and PEL93 cohorts were white, while brown skin color predominated in the SL97 cohort. Most subjects of the RP78 cohort lived with a partner, while most participants in the other three cohorts reported having no partner. Most participants belonged to class A/B in RP78 and RP94, while in PEL93 and SL97 the majority belonged to class C. At the time of the study, most participants in RP78, RP94 and PEL93 were not students. In the SL97 cohort, the majority was studying at the time of the interview. Except for SL97, most participants were working at the time of the interview in the RP78, RP94 and PEL93 cohorts. Significant differences in the distribution of the independent variables were observed between cohorts (Table 2).

**Table 2:** Distribution of the samples of the Ribeirão Preto (1978 and 1994), Pelotas (1993) and São Luís (1997) birth cohorts according to sociodemographic characteristics. RPS Consortium, Brazil.

Characteristics		Ribeirão Preto 1978	Ribeirão Preto 1994	Pelotas 1993	São Luís 1997	p
		% (95%CI)	% (95%CI)	% (95%CI)	% (95%CI)	
Sex		n=1,775	n=1,041	n=3,810	n=2,515	0.011*
	Male	47.7 (45.3–50.0)	41.9 (38.9–44.9)	46.8 (45.2–48.4)	47.6 (45.6–49.5)	
	Female	52.3 (50.0–54.6)	58.1 (55.1–61.1)	53.2 (51.6–54.8)	52.4 (50.5–54.4)	
Skin color†		n =1,769	n=1,040	n=3,437	n=2,500	0.001*
	White	79.1 (77.2–81.0)	75.8 (73.1–78.3)	65.8 (64.2–67.4)	19.8 (18.3–21.4)	
	Black	5.9 (4.9–7.1)	6.6 (5.2–8.3)	15.6 (14.5–16.9)	16.6 (15.2–18.1)	
	Brown	15.0 (13.4–16.7)	17.6 (15.4–20.0)	18.5 (17.3–19.7)	63.6 (61.6–65.4)	
Living with a partner		n=1,771	n=1,040	n=3,810	n=2,515	0.001*
	No	29.1 (27.0–31.2)	82.5 (80.0–84.7)	84.5 (83.3–85.6)	96.3 (95.5–97.0)	
	Yes	70.9 (68.7–73.0)	17.5 (15.3–19.9)	15.5 (14.4–16.7)	3.7 (3.0–4.5)	
Socioeconomic class		n=1,695	n=963	n=3,782	n=2,226	0.001*
	A/B	70.9 (68.7–73.0)	67.5 (64.5–70.4)	38.4 (36.8–39.9)	29.7 (27.8–31.5)	
	C	27.5 (25.4–29.7)	30.2 (27.4–33.2)	51.3 (49.7–52.9)	50.1 (48.1–52.2)	
	D/E	1.6 (1.1–2.3)	2.3 (1.5–3.4)	10.3 (9.4–11.3)	20.2 (18.6–21.9)	
Currently studying		n=1,771	n=1,040	n=3,810	n=2,515	0.001*
	No	89.5 (88.0–90.8)	53.6 (50.5–56.7)	64.9 (63.3–66.4)	30.5 (28.7–32.3)	
	Yes	10.5 (9.1–12.0)	46.4 (43.4–49.5)	35.1 (33.6–36.6)	69.5 (67.7–71.3)	
Currently working		n=1,610	n=798	n=3,567	n=2,515	0.001*
	No	7.7 (6.5–9.1)	19.3 (16.7–22.2)	32.7 (31.1–34.2)	84.2 (82.7–85.6)	
	Yes	92.3 (90.9–93.5)	80.7 (77.8–83.3)	67.3 (65.8–68.9)	15.8 (14.4–17.3)	

* p for the difference between cohorts;

† Indigenous and yellow subjects were excluded because of a small n.

Table 1 compares the prevalence of the outcomes between men and women of each cohort. At 37/39 years (RP78), there was a difference in the prevalence of insufficient sleep duration, insomnia and subjective sleep quality between men and women, with insufficient sleep duration being more frequent among men and insomnia and poor sleep quality among women. At 22 years, the differences between sexes were consistent in the RP94 and PEL93 cohorts, with insufficient sleep duration being more frequent among men and the other outcomes being more prevalent among women. The only exception was poor sleep quality in the RP94 cohort, which did not differ between sexes. At 18/19 years (SL97), insomnia, poor sleep quality and excessive sleepiness were more prevalent in women than in men. There was no difference in the prevalence of insufficient sleep duration or long latency between sexes at 18/19 years (Table 1).

### Subjective sleep quality and excessive daytime sleepiness among men

The variables most associated with the outcomes were studying and working at the time of the interview among participants aged 22 and 18/19 years (RP94, PEL93 and SL97). Being a student (80.6, 76.6 and 58.4% *versus* 65.2, 67.1 and 54.8% in the RP94, PEL93 and SL97 cohorts, respectively) or working (78.3, 75.3 and 60.7% *versus* 45.8, 55.3 and 53.1%) was associated with a higher prevalence of insufficient sleep in these three cohorts. Participants aged 22 years who did not study exhibited a higher prevalence of long latency in Pelotas (42.1 *versus* 36.8%) and a higher prevalence of insomnia in Ribeirão Preto (28.3 *versus* 19.9%). There was a higher prevalence of insomnia among subjects of the PEL93 cohort who did not work (28.8 *versus* 18.9%) and a higher prevalence of long latency among those of the SL97 cohort who did not work (37.1 *versus* 25.9%) (Supplementary material 1).

Skin color was associated with insomnia and excessive daytime sleepiness at 22 years in the PEL93 cohort, with the former being more frequent among brown subjects (29.3%) compared to whites (18.2%), and the latter among whites compared to brown subjects (59.6 *versus* 18.5%) (Supplementary material 2). Black skin color was associated with excessive sleepiness in the RP78 cohort (42.5%) compared to white skin color (22.0%). An association with the presence of a partner was only observed among men aged 37–39 years, with a higher prevalence of insufficient sleep among those living with a partner and of long latency and self-assessed poor sleep quality among those living without a partner. Poorer men (class D/E) more frequently exhibited insomnia (26.9 *versus* 18.8% among those of class A/B) and long latency (51.2 *versus* 37.4%) in Pelotas and more frequently had insomnia in São Luís (53.9 *versus* 45.9%) (Supplementary material 1).

### Subjective sleep quality and excessive daytime sleepiness among women

As observed for men, the characteristics associated with the largest number of outcomes were studying and working. Being a student was associated with insufficient sleep at 22 and 18/19 years and only with excessive daytime sleepiness at 22 years. Working was associated with insufficient sleep among women of the RP78 cohort (73.3 *versus* 60.7%) and among women from Pelotas (62.4 *versus* 49.0%). In the PEL93 cohort, daytime sleepiness was more frequent among subjects who worked (28.7 *versus* 22.0%). Not working was associated with long latency at 22 years in the two cohorts and with insomnia and poor sleep quality in women from Pelotas (Supplementary material 2).

An association with skin color was only observed in the PEL93 cohort, with a higher prevalence of long latency among brown women (56.5%) compared to white women (43.7%) and a higher prevalence of insomnia among black women (40.9%) compared to whites (30.0%) (Supplementary material 2). Women from Pelotas and young women from São Luís who lived without a partner more frequently had insufficient sleep and those of the RP78 cohort more frequently had long latency. Living without a partner was also associated with insomnia (33.5 *versus* 28.1%) and with excessive daytime sleepiness among women from Pelotas. In contrast, in RP94 and SL97, insomnia was more frequent among women living with a partner. Socioeconomic class D/E was associated with long latency in RP78 and with insomnia in PEL93. Young women from São Luís who belonged to class C more frequently had long latency (41.2%) and insomnia (58.1%) than those of class A/B (33.5 and 48.9%, respectively).

## DISCUSSION

The present study is the first to gather data on sleep, subjective sleep quality and excessive daytime sleepiness from different Brazilian birth cohorts using a similar method in different regions of the country. The study showed that insufficient sleep duration was the most frequent outcome among men and women of the four cohorts, whose prevalence was higher among men, except in SL97. Long latency was more frequent among young adult women of two cohorts than among men of the same age. Insomnia was more frequently reported by women than by men at the four ages investigated. Women generally suffered more from excessive sleepiness and evaluated their own sleep quality more negatively than men. In addition, being a student and working were associated with the largest number of outcomes in both sexes.

A previous study also identified a higher prevalence of long latency, terminal or maintenance insomnia, and self-assessed poor sleep quality among women^7^. On the other hand, insufficient sleep duration was more prevalent among men in that study^7^ and excessive daytime sleepiness was not associated with sex. According to the principles of biology, sleep fragmentation is greater in females, a fact that results in less continuous sleep when compared to males^17,18^. Another possible explanation might be the set of situations to which women are exposed, including the social demands related to work, family duties and esthetics, among other responsibilities assigned to them, which can result in unhealthy habits with negative impacts on sleep patterns^19^.

In males, insomnia was more frequent among brown participants and excessive daytime sleepiness among whites (RP94) and blacks (RP78). In females, there was a higher prevalence of long latency among brown women and of insomnia among black women (PEL93). A study involving university students aged 18 years or older found a higher prevalence of long latency (on class days) among brown and black students^5^. A study reported a longer latency time in African Americans^6^, which comprise brown and black skin color. However, the data were not stratified by sex in any of the cited studies. In the case of university students, a possible explanation suggested by the authors^5^ is sociodemographic and behavioral differences related to black or brown skin color, such as a lower social status and a larger number of individuals living in the same household^20^, which could affect the sleep pattern.

Regarding marital status, men (RP78) and women (RP94 and SL97) who lived with a partner exhibited a higher prevalence of insufficient sleep and insomnia, respectively. In contrast, the prevalence of long latency and self-assessed poor sleep quality was higher among men (RP78) who lived without a partner. Women who lived without a partner more frequently had insufficient sleep (PEL93 and SL97), long latency (RP78), insomnia and excessive daytime sleepiness (PEL93). The results regarding living with a partner agree with the study of Grandner et al.^8^, in which married couples were more likely to sleep less than those who were divorced, separated or widowed; and with the study of Chen et al.^21^, that showed higher levels of insomnia in married subjects compared to singles. These findings may be explained by higher levels of marital unhappiness^22^, although evidence indicates that the greater financial and social resources of married couples make them healthier, while unmarried people experience greater social isolation, a fact that increases stress^23^ and, consequently, the occurrence of sleep disorders.

Poorer men (class D/E) more frequently had insomnia (PEL93 and SL97) and long latency (PEL93); while long latency (RP78) and insomnia (PEL93) were more prevalent among poorer women. Young people from São Luís who belonged to class C more frequently had long latency and insomnia. In a study of a population-based sample of the city of Lausanne, Switzerland, with 3,391 participants aged 40/81 years, Stringhini et al.^7^ observed that both men and women with low socioeconomic status were more likely to have long sleep latency. Individuals of lower socioeconomic status may have more sleep disturbances because of problems in the disorganization of their home (noisier) and the presence of noise and other conditions unfavorable to sleep^24,25^, including a larger number of household members^20,25^. In addition, all the burdens and worries related to the conditions of life, work and insecurity of life, which can compromise the onset and maintenance of sleep and the perceived sleep quality.

Being a student was associated with a higher prevalence of insufficient sleep among men and women in RP94, PEL93 and SL97, and with a higher prevalence of excessive daytime sleepiness among women in RP94 and PEL93. Men who did not study had a higher prevalence of long latency (PEL93) and insomnia (RP94) when compared to those who study.

Changes in the sleep pattern of adolescents and young adults can be due to the obligations of school or academic life^26^, as well as the increase in social activities characteristic of this phase, which can lead to a reduction of sleep duration. The use of computers and other electronic devices can also reduce sleep duration and interfere with sleep quality^27^. It is important to note that some sleep problems have been considered a negative consequence or complication of internet addiction, common at this stage of life, which can cause anxiety, stress and depression in students^28^.

Working was associated with a higher prevalence of insufficient sleep in men (RP94, PEL93 and SL97) and women (RP78 and PEL93) and with excessive daytime sleepiness in women (PEL93). Men who did not work more frequently reported insomnia (PEL93) and long latency (SL97), while women more frequently reported long latency (RP94 and PEL93), insomnia and poor sleep quality (PEL93). In a study using data from the Campinas Municipal Health Survey (2014/15), Barros et al.^29^ found a difference in the prevalence of insomnia between adults and older adults who worked and those who did not, with a higher prevalence of the outcome in the latter. In Finland^9^, the prevalence of insomnia in adults was higher among those who had no occupation, regardless of age and sex. A possible explanation for this finding may be the lack of economic security and the social embarrassment resulting from unemployment, which can negatively affect mental health and, consequently, sleep characteristics^29^.

As limitations of the study, geographic differences in terms of racial distribution of the population, temperature and climate characteristics, health issues of each region, and regional access to health services, factors that were not investigated in the present study, may affect sleep disorders differently^30^. Furthermore, we cannot state that the differences found can be attributed to age or city of birth. Another limitation has to do with self-reported sleep characteristics, since the cutoff point used for short sleep is based on international studies.

As strengths of the study, we highlight that all surveys were carried out with high methodological rigor in the data collection and that the field staff was trained to reduce bias. In addition, the study includes the main birth cohorts of Brazil, which are located in three different regions with distinct demographic, socioeconomic and developmental characteristics. The size of the sample, which consisted of 9,141 subjects, must be emphasized, as well as the five sleep parameters that were estimated using the same instruments, permitting the analysis of prevalence and comparisons between cohorts.

In conclusion, this study showed that insufficient sleep duration was more frequent among men and long sleep latency was more frequent among young adult women (RP94 and PEL93). Insomnia was the problem most frequently reported by women in the four cohorts and women generally suffered more from excessive daytime sleepiness and evaluated their own sleep quality more negatively than men. In addition, being a student and working were the characteristics associated with the largest number of outcomes in both sexes.

These results should be interpreted with caution, as the findings are inconsistent, and we cannot suggest that attention needs to be directed to specific groups. We recommend a research agenda that examines the role sociodemographic characteristics play as disadvantages in sleep.
